# Autoimmune and Rheumatic Manifestations Associated With COVID-19 in Adults: An Updated Systematic Review

**DOI:** 10.3389/fimmu.2021.645013

**Published:** 2021-03-12

**Authors:** Kuo-Tung Tang, Bo-Chueh Hsu, Der-Yuan Chen

**Affiliations:** ^1^Division of Allergy, Immunology, and Rheumatology, Taichung Veterans General Hospital, Taichung, Taiwan; ^2^Faculty of Medicine, National Yang-Ming University, Taipei, Taiwan; ^3^Ph.D. Program in Translational Medicine and Rong Hsing Research Center for Translational Medicine, National Chung Hsing University, Taichung, Taiwan; ^4^Division of Allergy, Immunology and Rheumatology, Taichung Veterans General Hospital Puli Branch, Nantou, Taiwan; ^5^Translational Medicine Laboratory, China Medical University Hospital, Taichung, Taiwan; ^6^Rheumatology and Immunology Center, China Medical University Hospital, Taichung, Taiwan; ^7^College of Medicine, China Medical University, Taichung, Taiwan

**Keywords:** autoimmune disease, rheumatic disease, COVID-19, SARS-CoV-2, treatment

## Abstract

**Background:** Numerous cases of the coronavirus disease 2019 (COVID-19) with autoimmune and rheumatic manifestations have been reported. Despite the available reviews that summarized its autoimmune/rheumatic manifestations, a systematic approach is still lacking. Therefore, we conducted a comprehensive systematic review in order to give an overview upon these rare but clinically significant manifestations.

**Methods:** We performed a literature search of PubMed and EMBASE as of October 9, 2020. All articles relevant to either systemic or organ-specific autoimmune and rheumatic manifestations potentially associated with COVID-19 were collected. The reviewed literature were limited to adults ≥18 years.

**Results:** Although most of the existing evidence was based on case reports or case series without a long-term follow-up, a variety of autoimmune/rheumatic manifestations were associated with COVID-19. The manifestations that have a consistent association with COVID-19 include autoimmune cytopenia, cutaneous vasculitis, encephalitis, and Guillain-Barre syndrome. Such association is conflicting as regards to antiphospholipid syndrome, hemophagocytic lymphohistiocytosis, and myasthenia gravis.

**Conclusion:** Our systematic review indicated the potential of the COVID-19 virus to trigger a myriad of autoimmune and rheumatic manifestations, which should be considered amid global efforts to combat COVID-19.

## Introduction

Since the initial outbreak at Wuhan in December 2019, the coronavirus disease 2019 (COVID-19) pandemic has brought about a tremendous burden to the healthcare systems, and is still a huge threat to all human beings. As of 25th November 2020, nearly 60 million cases had been diagnosed, unfortunately with 1.4 million fatalities globally (1). Its manifestations ranged from asymptomatic infection, mild respiratory illness, acute respiratory distress syndrome (ARDS), and multiple organs failure. More and more reports regarding its associated autoimmune and rheumatic manifestations appeared as COVID-19 cases surged. These manifestations are noteworthy since they were either associated with increased morbidity, e.g., antiphospholipid antibody syndrome (APS), or life-threatening, e.g., multisystem inflammatory syndrome (MIS), as summarized in previous reviews (2, 3). Furthermore, one of the concerns about vaccination is its potential to cause similar autoimmune and rheumatic complications. With new reports accumulating at a rapid speed, we have undertaken a comprehensive systematic review and hoped it would help delineate the landscape of autoimmune and rheumatic manifestations associated with COVID-19.

## Materials and Methods

### Literature Search

**Figure 1:**
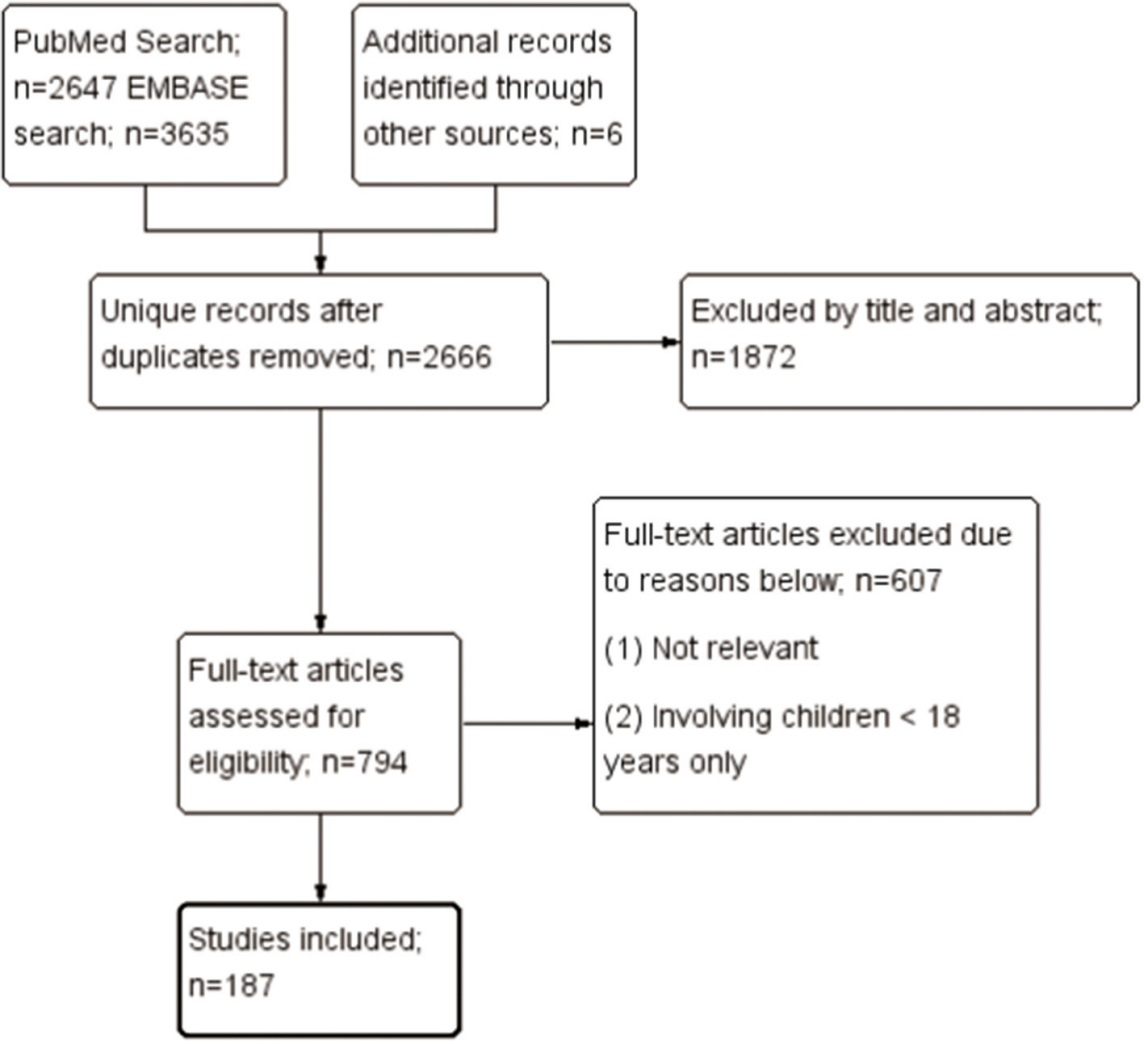
Selection of studies to be included in the systematic review.

This systematic review focused on the autoimmune and rheumatic manifestations associated with COVID-19 infection. The algorithm of the systematic review follows the Preferred Reporting Items for Systematic Reviews and Meta-Analyses (PRISMA) checklist, as shown in Figure 1. Firstly, we searched the PubMed and EMBASE on October 9, 2020. The search strategy is illustrated in detail in Appendix 1 in Supplementary Material. The search keywords for systemic autoimmune diseases included systemic lupus erythematosus (SLE), spondyloarthropathy, and hemophagocytic lymphohistiocytosis (HLH), etc.; those for organ-specific immune-related diseases included Guillain-Barré syndrome (GBS), uveitis, and interstitial lung disease (ILD), etc.

### Study Selection

Three authors (KT Tang, BC Hsu, and DY Chen) independently assessed the titles and abstracts identified by the aforementioned search, and the relevant full-text articles were retrieved. Two authors (KT Tang and DY Chen) independently assessed the full-text articles for eligibility, resolved discrepancies through discussion or consultation with the third author (BC Hsu). The references for the selected articles were also examined for relevance. Articles were selected if they: (1) involved adults ≥18 years; (2) were potentially relevant to autoimmune or rheumatic manifestations concurrent with or following COVID-19 infection; (3) were potentially relevant to an exacerbation of pre-existing autoimmune or rheumatic diseases concurrent with or following COVID-19 infection. There was no language restriction. Studies describing manifestations that were less likely immune-mediated were excluded, such as manifestations with onset apparently before the COVID-19 symptoms or those very likely medication-related. Two authors (KT Tang and DY Chen) independently extracted data from these studies electronically. Our emphasis was on the temporal relationship between these manifestations and COVID-19 infection, other clinical evidence supporting the immune-mediated mechanisms underlying these manifestations, the epidemiology of these manifestations, and therapeutic response to immunomodulating therapies.

## Results

### Overview

Probably owning to the characteristics of SARS-CoV-2 and the elicited immune response, COVID-19 infection has been reported to be associated with a variety of autoimmune and rheumatic manifestations. Most of these manifestations have been associated with other microbial infections and their underlying immune-mediated mechanisms are evident. However, the existing data were mostly case reports or case series, and sometimes even conflicting; therefore a causal relationship could not be ascertained.

### Potential Molecular Mechanisms Contributing to Autoimmune and Rheumatic Manifestations

#### Severe Acute Respiratory Syndrome Coronavirus 2 (SARS-CoV-2)

Coronaviruses contain the largest single-stranded RNA in nature, and the SARS-CoV-2 genome is composed of around 30,000 nucleotides (4). The complex transcriptome, due to its discontinuous transcription and recombination activities, may further expand the ability to interact with the immune system (5). Additionally, the variability of protein sequences provides a rich source of epitopes to stimulate the immune system (6). These factors may contribute to the development of immune-mediated manifestations associated with COVID-19 infection.

#### Molecular Mimicry

It is well-known that microbial infection could lead to autoimmunity through three mechanisms: molecular mimicry, bystander activation, and epitope spreading (7). The possibility of molecular mimicry in COVID-19 disease has been proposed, and peptide sharing analysis revealed massive hexapeptide and heptapeptide sharing between SARS-CoV-2 spike glycoprotein and human proteins compared with other mammals and human coronaviruses (8). Another study also found hexapeptide sharing between viral epitopes and 460 human proteins (9). Interestingly, some of these proteins are associated with pulmonary, cardiac, vascular, coagulation, and immunological disorders. Other studies have demonstrated a similarity between SARS-CoV-2 and human proteins, including pulmonary surfactants (10), brainstem neuronal proteins (11), chaperons (12), heat shock proteins 60 and 90 (13), ankyrin 1 (an erythrocyte membrane protein) (14), odorant receptor 7D4, poly (ADP-ribose) polymerase family member 9 (PARP9), and solute carrier family 12 member 6 (SLC12A6) (15), which have been hypothesized to contribute to lung disease, respiratory failure, endothelitis, neuroimmune diseases, autoimmune hemolytic anemia (AIHA), leukopenia, and vascular damage. Also, three immunogenic epitopes with high sequence identity to viral proteins were found in patients with dermatomyositis (16). Of note, the monoclonal antibodies against SARS-CoV-2 spike protein and nucleoprotein could cross-react with various tissue proteins, such as nuclear antigens, extractable nuclear antigen, mitochondria, thyroglobulin, thyroid peroxidase, transglutaminases, myelin basic protein, actin, and α-myosin (17). In summary, molecular similarities between viral and human proteins existed, but the clinical significance requires further verification.

#### Autoantibodies

As shown in Table 1, five studies reported the screening results of circulating autoantibodies in patients with COVID-19 (17–21). Anti-nuclear antibodies (ANA) was found in 4–50% of COVID-19 patients, most of whom were older people. This was consistent with previous reports of an increased prevalence of autoantibodies in the elderly without autoimmune diseases (22). In accordance, Schiaffino et al. found an association between older age and the presence of autoantibodies in COVID-19 patients (17). Preliminary results of these studies indicated a higher incidence of neurologic and thrombotic events, and poor outcome in the autoantibody-positive subgroup compared with the autoantibody-negative subgroup (17, 18). However, the pathogenic potential of these autoantibodies, and whether these autoantibodies persist after resolution of COVID-19 infection, remains unclear. Interestingly, Bastard et al. demonstrated the presence of anti-type I interferon (IFN) antibodies in 10.2% of 987 patients with life-threatening COVID-19 pneumonia, although they speculated that these antibodies might appear before COVID-19 infection (23). In summary, autoantibodies were prevalent in COVID-19 patients, albeit with unknown clinical significance.

**Table 1:** Prevalence of circulating autoantibodies in patients with COVID-19 disease.

**Study**	**Reference number**	**Country**	**Patients**	**Mean/median age (years)**	**Proportion of males**	**Autoantibodies**
Pascolini et al.	18	Italy	33 referred patients	70 (range 22–90)	52%	ANA detected by IFA on HEp-2 cells (33%), anti-histone antibody detected by immunoblot (3%), but negative for autoantibodies against Sm and RNP/Sm, RNP70, A, and C, SSA-Ro52, SSA-Ro60, SSB, Scl-70, PM-Scl, Jo-1, CENP-B, PCNA, dsDNA, nucleosomes, ribosomal P protein, and M2 detected by immunoblot, ANCA detected by IFA, and anti-PR3 and anti-MPO antibodies detected by FEIA
Schiaffino et al.	19	Spain	53 hospitalized patients	64 (IQR 24–91)	58%	ANA detected by unknown method (3.8%), IgG/M autoantibodies against hepatocytes and gastric glandular cells detected by IFA on rat kidney/stomach/liver (23%)
Vlachoyiannopoulos et al.	20	Greece	29 ICU patients	64 (range 43–85)	72%	ANA detected by unknown method (34.5%), anti-CCP detected by ELISA (3.5%), c-ANCA detected by immunofluorescence (6.9%), and p-ANCA detected by immunofluorescence (6.9%), but negative for anti-ENA detected by immunoblot, and anti-dsDNA, anti-PR3 and anti-MPO antibodies detected by ELISA
Vojdani et al.	17	USA	5 patients	N.A.	N.A.	ANA, anti-ENA, anti-actin and anti-mitochondrial antibodies detected by unknown methods (60%), but negative for anti-dsDNA antibody and RF detected by unknown methods
Zhou et al.	21	China	21 ICU patients	66 (SD 13)	62%	ANA (50%), anti–Ro52 (20%), anti–Ro60 (25%), anti-Scl-70 (5%), and anti-U1-RNP antibodies (5%), but negative for autoantibodies against Jo-1, centromere B, SmD1, SSB and dsDNA (all detected by chemiluminescence immunoassay)

*ANA, antinuclear antibody; CCP, cyclic citrullinated peptide antibody; COVID-19, the coronavirus disease 2019; c-ANCA, cytoplasmic anti-neutrophil cytoplasmic antibody; dsDNA, double-stranded DNA; ELISA, enzyme-linked immunosorbent assay; ENA, extractable nuclear antigen; FEIA, fluorescent-enzyme immuno-assay; ICU, intensive care unit; IFA, indirect immunofluorescence assay; IQR, interquartile range; MPO, myeloperoxidase; N.A., not available; p-ANCA, perinuclear anti-neutrophil cytoplasmic antibody; PCNA, proliferating cell nuclear antigen; PR3, proteinase 3; RF, rheumatoid factor; RNP, ribonucleoprotein; SD, standard deviation*.

#### Cytokine Storm

COVID-19 triggers an exaggerated immune response in infected patients, and a variety of inflammatory cytokines, such as interleukin (IL)-1β, IL-6, IL-8, interferon (IFN)-γ, and chemokines, such as granulocyte colony stimulating factor (G-CSF), interferon gamma-induced protein 10 (IP-10), monocyte chemoattractant protein-1 (MCP-1), and macrophage inflammatory protein 1α (MIP-1α), were elevated in severe COVID-19 patients (24, 25). In particular, a meta-analysis has demonstrated a nearly 3-fold higher serum levels of IL-6 in patients with complicated COVID-19 when compared with those patients with non-complicated disease (26). Regulatory T cells were also below normal levels in COVID-19 patients, which further aggravate the inflammatory response (27). Ultimately, the resultant cytokine storm leads to tissue damage and multiple organ failure. Clinically, inflammatory markers, such as C-reactive protein, procalcitonin, D-dimer, and ferritin, were increased in COVID-19 patients and associated with a poor prognosis (28). Taken together, the uncontrolled inflammatory milieu triggered by COVID-19 infection may lead to organ damage and the generation of autoimmunity, too.

### Systemic Autoimmune and Rheumatic Manifestations

#### Arthritis

Articular symptoms are often observed in virus infection, with the severity ranging from arthralgia, acute arthritis, to chronic arthritis (29). Only two studies reported the prevalence of arthralgia alone, instead of myalgia/arthralgia, in COVID-19 patients, which were 31% of 417 patients with COVID-19 from 12 European hospitals and 2.5% of 40 patients in Thailand, respectively (30, 31). Five cases of acute mono-, oligo-, or polyarthritis as an initial presentation (32) or a delayed phenomenon 3–29 days after COVID-19 symptoms onset (33–36) have been reported. Two cases had accompanying features of reactive arthritis, such as enthesitis and urethritis (35, 36). Using RT-PCR, negative SARS-CoV-2 RNA in joint fluid was demonstrated in two cases (33, 36), implying that the arthritis was mediated by immune mechanisms rather than direct viral invasion. Acute arthritis either resolved spontaneously (33) or responded to treatment with non-steroidal anti-inflammatory drugs (NSAIDs), corticosteroids, and even baricitinib (32, 34–36). Nevertheless, two acute arthritis patients did not enter remission at follow-up despite treatment (32, 35). Furthermore, insufficient follow-up time in these reports might miss out on the opportunity to observe arthritis recurrences after arthritis remission and being drug-free, since infection-related reactive arthritis may persist for years (37). Development of chronic arthritis concurrent with SARS-CoV-2 infection was found in a 45-year-old male, which responded to corticosteroids (38). Another 50-year-old female also demonstrated worsening of pre-existing rheumatoid arthritis (RA), which improved after sarilumab treatment (32). In summary, SARS-CoV-2 infection was associated with the development of arthralgia, acute arthritis, and possibly, chronic arthritis.

#### Antiphospholipid Antibody Syndrome (APS)

**Figure 2:**
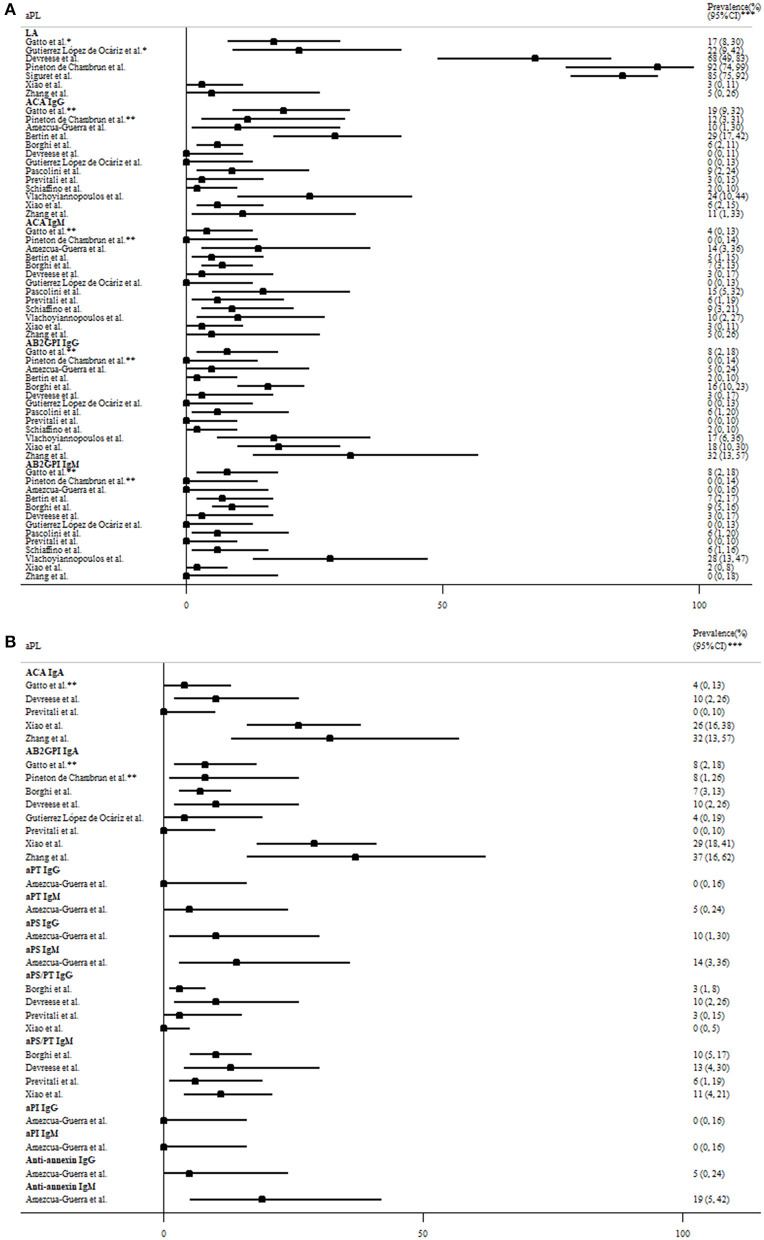
The prevalence of **(A)** criteria and **(B)** non-criteria antiphospholipid antibodies (aPL) based on the revised Sapporo criteria for antiphospholipid antibody syndrome in COVID-19 patients with moderate to severe disease. *Determined by two tests based on different principles per the International Society of Thrombosis and Haemostasis criteria. **Moderate-to-high titer aPL. ***95% exact confidence intervals. AB2GPI, anti-β2glycoprotein I antibody; ACA, anticardiolipin antibody; aPI, antiphosphatidylinositol antibody; aPL, antiphospholipid antibodies; aPS, antiphosphotidylserine antibody; aPT, antiprothrombin antibody; COVID-19, the coronavirus disease 2019; LA, lupus anticoagulant.

As demonstrated in Table 2, the presence of antiphospholipid antibodies (aPL) has been observed in COVID-19 patients around the world (18–20, 39–55). The association between aPL and disease severity was shown in three studies (39, 54, 56), but not in another study (46). Lupus anticoagulant and non-criteria IgA anti-β2glycoprotein-I/anticardiolipin antibodies (57) were the most prevalent aPL, with the prevalence of 3–92, 0–37, and 0–32% in patients with moderate to severe disease (Figure 2). However, the lupus anticoagulant testing might be interfered by heparin use or elevated C-reactive protein in COVID-19 patients. IgG anticardiolipin and anti-β2glycoprotein-I antibodies were also prevalent, but often in low titers. Notably, one study revealed 3 (5%) of 58 COVID-19 patients had a highly thrombogenic anti-β2glycoprotein-I domain I IgG antibody, although not correlated with thrombosis (41). The strong association of aPL with thrombotic events was not observed in most studies, even for patients with double/triple positivity. Besides, repeated testing showed that the titer of aPL fluctuated during the disease course (52, 54), and the aPL turned to be negative 1 month later in most of the aPL-positive patients (44).

**Table 2:** Prevalence of antiphospholipid autoantibodies in COVID-19 disease.

**Study**	**Reference number**	**Country**	**Patients**	**Mean/median age (years)**	**Proportion of males**	**Prevalence of aPL**	**Findings**
Amezcua-Guerra et al.	39	Mexico	21 ICU patients	62 (IQR 54–67)	43%	Anti-annexin V IgG (5%), anti-annexin V IgM (19%), ACA IgG (10%), ACA IgM (14%), AB2GPI IgG (5%), AB2GPI IgM (0%), aPT IgG (0%), aPT IgM (5%), aPS IgG (10%), aPS IgM (14%), aPI IgG (0%), and aPI IgM (0%)*	Elevated levels of interleukin-6/ferritin/C-reactive protein only in patients with aPL; pulmonary embolism in two aPL+ patients but in no aPL- patients
Bertin et al.	40	France	56 patients with moderate and severe disease	67	59%	ACA IgG (29%), ACA IgM (5%), AB2GPI IgG (2%), and AB2GPI IgM (7%)*	ACA IgG was associated with severe disease
Borghi et al.	41	Italy	122 patients with severe disease	69 (SD 16)	63%	ACA IgG (6%), ACA IgM (7%), AB2GPI IgG (16%), AB2GPI IgG domain I (5%), AB2GPI IgM (9%), AB2GPI IgA (7%), aPS/PT IgG (3%), and aPS/PT IgM (10%)*	No association between aPL and thrombotic events, even for AB2GPI domain I IgG
Bowles et al.	42	UK	35 patients with a prolonged aPTT	57 (95%CI 19–83)	69%	LA (53%)**	
Cuenca Saez et al.	43	Spain	11 patients with perniosis	(range 2–40)	N.A.	LA (0%), ACA IgG (0%), ACA IgM (0%), and low titer ACA IgA (100%)	
Devreese et al.	44	Belgium	31 ICU patients	63 (range 38–82)	90%	LA (68%), ACA IgG (0%), ACA IgM (3%), ACA IgA (10%), AB2GPI IgG (3%), AB2GPI IgM (3%), AB2GPI IgA (10%), aPS/PT IgG (10%), and aPS/PT IgM (13%)	No association between aPL and thrombotic events
Galeano-Valle et al.	45	Spain	24 patients with venous thromboembolism	64 (SD 14)	58%	ACA IgG (0%), low titer ACA IgM (8.3%), AB2GPI IgG (0%), and low titer AB2GPI IgM (8.3%)	
Gatto et al.	46	Italy	122 patients with mild to severe disease	54 (SD 19)	49%	LA (22%)**, ACA IgG (13%), ACA IgM (3%), ACA IgA (2%), AB2GPI IgG (6%), AB2GPI IgM (7%), and AB2GPI IgA (3%)	A trend toward an association between aPL and thrombotic events
Gutierrez López de Ocáriz et al.	47	Spain	27 hospitalized patients	58 (range 20–90)	44%	LA (22%)**, ACA IgG (0%), ACA IgM (0%), AB2GPI IgG (0%), AB2GPI IgM (0%) and AB2GPI IgA (4%)*	No association between aPL and thrombotic events
Harzallah et al.	48	France	56 patients	N.A.	N.A.	LA (45%)** and ACA IgG/M/AB2GPI IgG/M (10%)	
Pascolini et al.	18	Italy	33 hospitalized patients	70 (range 22–90)	52%	ACA IgG (9%), ACA IgM (15%), AB2GPI IgG (6%), and AB2GPI IgM (6%)*	None of the patients had thrombotic events
Pineton de Chambrun et al.	49	France	25 ICU patients	48 (range 35–64),	68%	LA (92%), ACA IgG (12%), ACA IgM (0%), ACA IgA (8%), AB2GPI IgG (0%), AB2GPI IgM (0%), and AB2GPI IgA (8%)	Massive pulmonary embolism in 6 patients, all aPL+
Previtali et al.	50	Italy	35 deceased patients	73 (range 52–82)	74%	Low titer ACA IgG (3%), low titer ACA IgM (6%), ACA IgA (0%), AB2GPI IgG (0%), AB2GPI IgM (0%), low titer aPS/PT IgG (3%), and low titer aPS/PT IgM (6%)	Catastrophic APS was less likely despite multiple thrombosis at autopsies
Reyes Gil et al.	51	USA	68 patients	57	50%	LA (60%)**, ACA IgG (0%), ACA IgM (1%), AB2GPI IgG (0%), and AB2GPI IgM (1%)*	LA associated with thrombotic events
Schiaffino et al.	19	Spain	53 hospitalized patients	64 (range 24–91)	58%	ACA IgG (2%), ACA IgM (9%), AB2GPI IgG (2%), and AB2GPI IgM (6%)*	No association between aPL and thrombotic events
Siguret et al.	52	France	74 mechanically ventilated patients	64	N.A.	LA (85%) and ACA IgG/IgM/AB2GPI IgG (12%)*	No association between aPL and thrombotic events
Tvito et al.	53	Israel	43 patients with mild to severe disease	N.A.	63%	LA (37%)**, ACA IgG (0%), ACA IgM (0%), AB2GPI IgG (0%), and AB2GPI IgM (0%)	No association between aPL and thrombotic events
Vlachoyiannopoulos et al.	20	Greece	29 ICU patients	64 (range 43–85)	72%	ACA IgG (24%), ACA IgM (10%), ABGPI IgG (17%), and ABGPI IgM (28%)*	
Xiao et al.	54	China	66 ICU patients	65	59%	LA (3%), ACA IgG (6%), ACA IgM (3%), ACA IgA (26%), AB2GPI IgG (18%), AB2GPI IgM (2%), AB2GPI IgA (29%), aPS/PT IgG (0%), and aPS/PT IgM (11%)*	Patients with multiple aPLs had a significantly higher incidence of cerebral infarction
Zhang et al.	55	China	19 ICU patients	65 (IQR 60–70)	53%	LA (5%), ACA IgG (11%), ACA IgM (5%), ACA IgA (32%), AB2GPI IgG (32%), AB2GPI IgM (0%), and AB2GPI IgA (37%)*	All 4 patients with cerebral infarction had aPL with multiple isotypes whereas no thrombotic events developed in aPL-patients.

* *Probably including low titer aPL as positive*.

** *Determined by two tests based on different principles per the International Society of Thrombosis and Haemostasis criteria*.

*AB2GPI, anti-β2glycoprotein I; ACA, anticardiolipin antibody; aPI, antiphosphatidylinositol antibody; aPL, antiphospholipid antibodies; aPS, anti-phosphotidylserine antibody; aPT, antiprothrombin antibody; aPTT, activated partial-thromboplastin time; CI, confidence interval; COVID-19, the coronavirus disease 2019; ICU, intensive care unit; IQR, interquartile range; LA, lupus anticoagulant; N.A., not available; SD, standard deviation*.

COVID-19 was associated with the flares of pre-existing APS. The complications include bilateral adrenal glands hemorrhage in a 66-year-old female and limb ischemia in another 48-year-old male, both of which were controlled by anticoagulants (58, 59). Interestingly, one study revealed 5 (63%) of eight pregnant women with COVID-19 infection fulfilled diagnostic criteria for pre-eclampsia/HELLP syndrome (hemolysis; elevated liver enzymes; low platelet count), perhaps due to overlapping features. Only one of them was more likely to have pre-eclampsia, an obstetric complication of APS (60). However, aPL were not examined in these pregnant women. In summary, low titer and transient aPL were prevalent in COVID-19 patients, but like aPL generated in other infections, most of them were not pathogenic.

#### Multisystem Inflammatory Syndrome in Adults (MIS-A)

Pediatric cases of Kawasaki-like multisystem inflammatory syndrome (MIS-C) are accumulating mainly in Western countries. With a favorable prognosis, the disease was characterized by hyper-inflammation, gastrointestinal symptoms, and cardiac dysfunction, such as myocarditis, and shock, which were somewhat different from classical Kawasaki disease. Multisystem inflammatory syndrome associated with COVID-19 infection has also been found in adults (MIS-A) aged 35–54 years (61–64). Like children, these adult patients were reported in Western countries and recovered after treatment with corticosteroids, intravenous immunoglobulin (IVIG), and tocilizumab, an IL-6 receptor inhibitor. An IL-1 receptor antagonist, anakinra, may also be a therapeutic option based on the experiences in children (65). Although some did not undergo complete coronary evaluation, none of these adult patients had dilatation or aneurysm. Interestingly, isolated myocarditis has also been reported in two adult COVID-19 patients: one 28-year-old woman presented with myocarditis shortly after COVID-19 infection, which improved after methylprednisolone pulse therapy (66); the other 53-year-old woman developed myocarditis a week after COVID-19 symptoms onset, which was stabilized by corticosteroids (67).

#### Systemic Vasculitis

An autopsy study in Italy revealed vasculitis in the lung, brain, and other organs in individuals who succumbed to COVID-19 (68). Clinical evidence for systemic vasculitis was limited to case reports. A 69-year-old woman and a 71-year-old man presented with asymptomatic aortitis and had concurrent SARS-CoV-2 infection (69, 70). A 73-year-man presented with arterial vasculitis at splenic hilum resulting in splenic infarction concomitantly with COVID-19 infection. The condition improved after splenectomy (71). Another 71-year-old man developed abdominal and bilateral common iliac arteritis concurrent with COVID-19 infection, with subsequent spontaneous remission (70). Concurrent ileocecal vasculitic ulcers was found in a 40-year-old female COVID-19 patient, who then only received supportive treatment (72). Notably, virus particles have been found in the cytoplasm of vascular endothelial cells in the biopsy specimen of one patient (72), whereas SARS-CoV-2 RT-PCR was negative in another patient's biopsy (71). A 37-year-old woman suffered from anti-proteinase 3 (PR3)-positive diffuse alveolar hemorrhage concurrent with SARS-CoV-2 infection and later received treatments of intravenous methylprednisolone pulse therapy, plasmapheresis, and IVIG. Her hemoptysis improved after IVIG, but finally she expired while on ventilator (73). Two cases were reported on anti-neutrophilic cytoplasmic antibodies (ANCA)-associated vasculitis with necrotizing nephritis concomitant with COVID-19 infection. They responded to methylprednisolone pulse therapy plus rituximab (74). Henoch-Schönlein purpura with nephritis was found in a 78-year-old man 3 weeks after COVID-19 infection. His condition improved after methylprednisolone pulse therapy and rituximab (75). Henoch-Schönlein purpura with suspected gastrointestinal involvement occurred in another 24-year-old man, and improved after corticosteroids treatment (76). In summary, case reports of large, medium, and small vessel vasculitis involving multiple organs have been reported in COVID-19 patients. However, we could not ascertain whether these vasculitis resulted from direct virus-induced endothelitis or immune-mediated mechanisms.

#### Other Systemic Autoimmune Rheumatic Diseases (SARDs)

Three cases of new onset SLE concurrent with COVID-19 infection have been published, and two of them eventually deceased (77–79). In other studies, myositis of proximal limbs and paraspinal myositis have been demonstrated on magnetic resonance imaging (MRI) along with elevated creatine kinase in one and seven COVID-19 patients, respectively (80, 81). However, a complete workup, such as electromyography and muscle biopsy, was lacking in these cases. None of them received treatment due to being either in critical conditions or asymptomatic. In a Chinese cohort of 21 COVID-19 patients with pre-existing SARDs, a disease flare was demonstrated in one SLE patient (skin rashes and hemolytic anemia), one ankylosing spondylitis patient (back and ankle pain), and one patient with polymyalgia rheumatica (muscle pain), whose symptoms were attenuated after treatment with hydroxychloroquine, NSAID, corticosteroids, or mycophenolate mofetil (82). Another Italian multicenter cohort reported 40 (17%) of 232 SARD patients with moderate to severe disease activity upon COVID-19 infection but provided no further details (83). The Asia Pacific Lupus Collaboration (APLC) cohort reported three cases of SARS-CoV-2-infected SLE patients, two of whom developed a concurrent lupus flare (thrombocytopenia and nephritis) and improved after corticosteroids and IVIG treatment (84). Another two SARS-CoV-2-infected SLE patients presented with an exacerbation of thrombocytopenia, which were successfully treated with corticosteroids and IVIG (85, 86). In summary, SARDs, especially SLE, might flare upon COVID-19 infection. Nevertheless, the overlapping features between the wide spectrum of COVID-19 manifestations and SARDs made the distinction ambiguous. Furthermore, medication adherence in these patients was questionable during the pandemic, which might also contribute to a flare of SARDs.

#### Hemophagocytic Lymphohistiocytosis (HLH)

Infection is a well-known trigger factor of HLH. In fact, severe COVID-19 patients presented with hyperferritinemia and cytokine storm, reminiscent of HLH. Autopsy studies demonstrated a high percentage of hemophagocytosis in COVID-19 patients' bone marrow, pulmonary lymph nodes, or spleen, ranging from 75 to 94% (87, 88). Using a validated hemophagocytic syndrome diagnostic score (HScore) cut-off point of 168 (89), we identified a total of 15 cases of COVID-19-associated HLH (Table 3) (88, 90–94). However, the reported prevalence of HLH in severe COVID-19 patients was as low as 7% (93, 94). Notably, routine examinations for serum triglycerides and fibrinogen, hepatosplenomegaly, or tissue hemophagocytosis were not always performed in these studies. In summary, COVID-19 infection was associated with HLH in a few cases as defined by the HScore.

**Table 3:** Hemophagocytic lymphohistiocytosis (HLH) in COVID-19 disease.

**Study**	**Reference number**	**Country**	**Patient number**	**Age (years), median (range)**	**Sex**	**HSscore, median (range)**	**Treatment response**
Debliquis et al.	90	France	1	63	1M	207	Deceased without specific treatment
Dimopoulos et al.	91	Greece and the Netherlands	8	68 (51, 84)	7M1F	175 (171, 188)	Decreased HScore after anakinra but 3 (38%) of them eventually deceased
Faguer et al.	92	France	1	51	1M	253	Decreased HScore after tocilizumab
Hakim et al.	93	USA	1	37	1M	204	Decreased HScore after tocilizumab but eventually deceased on ventilator
Prilutskiy et al.	88	USA	1	72	1M	217	Hemophagocytosis found post-mortem despite anakinra
Wood et al.	94	UK	3	N.A.	N.A.	N.A.	Decreased HScore after tocilizumab but then contracting a bacterial pneumonia

*COVID-19, the coronavirus disease 2019; N.A., not available*.

### Organ-Specific Immune-Related Manifestations

#### Hematological Manifestations

Thrombocytopenia is common but usually mild in COVID-19 infection. Chen et al. reviewed 271 hospitalized COVID-19 patients and found the prevalence of delayed thrombocytopenia 14 days after COVID symptoms to be 12%. The authors speculated that it was partly immune-mediated (95). Table 4 demonstrated 38 cases of immune thrombocytopenic purpura (ITP) associated with COVID-19 infection, and some of them only had mild or asymptomatic COVID-19 (90, 96–113). Most of these patients with thrombocytopenia were diagnosed by exclusion and had a favorable response to corticosteroids, IVIG, and even thrombopoietin receptor agonists. Positive direct Coomb's test was shown in 13% of 267 anemic COVID-19 patients and 46% of the other 113 COVID-19 patients (114, 115). Furthermore, both warm and cold AIHA have been reported in COVID-19 patients and most of them recovered spontaneously (116–122). In line with these findings, a 39-year-old man was found to have concomitant Evans syndrome and COVID-19 infection (123). Autoimmune thrombotic thrombocytopenic purpura has been demonstrated in two COVID-19 patients and improved after plasmapheresis (124, 125). Another 66-year-old man whose acquired hemophilia flared concomitantly with COVID-19 infection, responded to corticosteroids and cyclophosphamide (126). In summary, many cases of ITP or AIHA associated with COVID-19 have been reported, implying a possible link between them.

**Table 4:** Cases of immune thrombocytopenic purpura (ITP) in COVID-19 disease.

**Study**	**Reference number**	**Age (years)**	**Patient number and sex**	**Findings**	**Treatment response**
Artru et al.	96	38	1M		Responsive to corticosteroids and IVIG
Bennett et al.	97	73	1F		Responsive to methylprednisolone pulse therapy and IVIG
Bomhof et al.	98	59, 66, 67	2M1F		Two responsive to corticosteroids and IVIG, and one died of intracerebral bleeding despite platelet transfusion
Debliquis et al.	90	78	1M	Increased megakaryocytes at bone marrow biopsy, positive antiplatelet antibodies	Responsive to IVIG
Deruelle et al.	99	41	1M	Increased megakaryocytes at bone marrow biopsy	Responsive to IVIG
Hindilerden et al.	100	86	1M	Increased megakaryocytes at bone marrow biopsy	Responsive to corticosteroids
Hu et al.	101	72	1F	History of ITP	Responsive to corticosteroids
Humbert et al.	102	84	1M		Responsive to corticosteroids and IVIG
Lévesque et al.	103	53	1M		Responsive to romiplostim, vincristine, and methylprednisolone pulse therapy
Mahevas et al.	104	Median 64 (range 53–79)	7M7F		All responsive to corticosteroids and IVIG
Malik et al.	105	29	1F	Increased megakaryocytes at bone marrow biopsy	Responsive to corticosteroids
Martincic et al.	106	48	1M		Responsive to corticosteroids and IVIG
Murt et al.	107	41	1M		Responsive to IVIG
Nesr et al.	108	34	1F	History of ITP, pregnant	Responsive to corticosteroids and IVIG
Pascolini et al.	109	31, 69, and 88	2M1F	Positive IgM antiplatelet antibodies	Recovery after resolution of COVID-19 infection
Patel et al.	110	67	1M		Responsive to romiplostim
Revuz et al.	111	39, 57, and 76	2M1F		All responsive to IVIG
Sadr et al.	112	57	1F		Recovery after resolution of COVID-19 infection
Zulfiqar et al.	113	65	1F	Increased megakaryocytes at bone marrow biopsy	Responsive to corticosteroids and eltrombopag

*COVID-19, the coronavirus disease 2019; IVIG, intravenous immunoglobulin*.

#### Skin Manifestations

Case series had been reported in Western countries on Raynaud's phenomenon and chilblains-like lesions in patients with recent COVID-19 infection (positive anti-SARS-CoV-2 IgG) or in close contact with confirmed COVID-19 cases (127–130). Some biopsies revealed positive findings for vasculitis or vascular microthrombi, but negative findings for SARS-CoV-2 based on RT-PCR. Biopsy-proven cases had been reported on cutaneous vasculitis, manifesting as purpuric papules (131) and plaques (132, 133), hemorrhagic bullae (134), and urticarial (135, 136), and targetoid lesions (137). Most of these conditions occurred 5–35 days after onset of COVID-19 symptoms and they responded well to topical or oral corticosteroids. Besides, livedo reticularis developed without aPL in a 57-year-old man concomitant with COVID-19 infection (138). Flares of psoriasis or newly-developed psoriatic arthritis concurrent with or following COVID-19 infection had been reported. These conditions had resolved either spontaneously or after NSAID and topical corticosteroids treatment (139–141). In summary, cutaneous vasculitis were potentially associated with COVID-19 infection, mainly in the Western population.

#### Neurological Manifestations

A spectrum of neurological manifestations, including neuroimmune manifestations were presented in COVID-19 patients. The frequencies of these manifestations in hospitalized patients are as follows: encephalitis 0.1–0.2%, GBS 0.1–1%, myelitis 0.1%, and optic neuritis 0.1% in Western countries (142–144). In Singapore, two cases of acute disseminated encephalomyelitis (ADEM), two encephalitis, and one GBS were reported among 47,572 COVID-19 cases (145). The development of encephalitis and GBS were generally delayed after COVID-19 symptoms onset. MRI-proven central nervous system vasculitis occurred in two patients 11–29 days after COVID-19 symptoms onset and responded to either corticosteroids or tocilizumab (146, 147). Using RT-PCR or antibody testing, if available, cerebrospinal fluid SARS-CoV-2 were positive in only some of these cases (144). Most neuroimmune diseases patients responded to standard immunomodulating therapies.

A 29-year-old woman developed multiple sclerosis with right optic neuritis 2–3 weeks after COVID-19 infection (148). Another 26-year-old man had myelin oligodendrocyte glycoprotein antibody-positive neuromyelitis optica, presenting as bilateral optic neuritis and longitudinal extensive transverse myelitis a few days after COVID-19 symptoms onset and responsive to methylprednisolone pulse therapy (149). Also, a study of 76 patients with multiple sclerosis demonstrated disease recrudescence preceding or concurrent with COVID-19 in 16 (21%) (150). There were reports of newly-onset acetylcholine receptor antibody-positive myasthenia gravis (MG) 5–7 days after COVID-19 symptoms onset, which responded to pyridostigmine, corticosteroids, IVIG and plasmapheresis (151). In a cohort of 15 MG patients with concomitant SARS-CoV-2 infection, a high percentage of them experienced MG worsening (87%) and even mechanical ventilation (73%), although it was difficult to ascertain exacerbated respiratory muscle weakness in patients with concomitant pneumonia and respiratory failure (152). However, 8 (62%) of them recovered after treated with corticosteroids, IVIG or plasmapheresis. In another cohort, one (20%) of 5 SARS-CoV-2-infected MG patients experienced MG worsening (ptosis and dysphagia) but responded to corticosteroids plus IVIG (153). In summary, a myriad of neuroimmune manifestations might develop concurrently with or following SARS-CoV-2 infection. However, it was difficult to differentiate between direct neuronal infection and immune-mediated mechanisms underlying these manifestations.

#### Interstitial Lung Disease (ILD)

Lung involvement is critical in COVID-19 infection. Chest computed tomography (CT) demonstrated interlobular septal and interstitial thickening in 0.88% of 130 infected inpatients at Wuhan (154). In accordance, autopsy of COVID-19 patients with critical illness showed interstitial mononuclear inflammatory infiltrates, organizing pneumonia, or fibrosis in the lung (155, 156). Long-term follow-ups of SARS survivors had residual fibrosis on chest radiographs and accompanying respiratory dysfunction 6 months later (157). Similarly, a subset of COVID-19 patients displayed interstitial change 2 weeks after disease onset (158) or upon discharge (159), as well as decreased diffusing capacity for carbon monoxide (DLCO) (160). However, a longer follow-up is required to monitor the progression of pulmonary interstitial change after COVID-19 infection to determine if these changes are merely post-ARDS change or progressive ILD.

#### Ocular Manifestations

Retinal vein vasculitic occlusion was found in a 52-year-old man 10 days after COVID-19 symptoms appeared, but his visual acuity improved after treatment with corticosteroids and intravitreal anti-vascular endothelial growth factor (anti-VEGF) injection (161). Another 54-year-old woman developed bilateral anterior uveitis 14 days after COVID-19-associated MIS-A but responded to topical corticosteroids (61).

#### Other Organ-Specific Immune-Related Manifestations

Crescentic glomerulonephritis had been found in two COVID-19 patients, which stabilized after methylprednisolone pulse therapy, plasmapheresis, IVIG and cyclophophomide (162). Collapsing glomerulopathy concomitant with COVID-19 infection was reported in two cases, with virus-like particles found in their renal biopsies (163, 164). In the North West London, eight unexpected new cases of Goodpasture syndrome were diagnosed, representing a 5-fold increase of the background rate, and four of them were positive for IgM and/or IgG antibodies to SARS-CoV-2 (165). However, the clinical significance required further research. A 19-year-old woman developed ulcerative colitis 9 days after the resolution of COVID-19 infection (166). A cross-sectional study of 82 patients with inflammatory bowel disease (IBD) revealed an IBD flare in one (1%) patient during SARS-CoV-2 infection (167). In another study of 79 IBD patients infected with SARS-CoV-2 revealed 4 (5%) patients had severe concomitant IBD flare (168). Finally, subacute thyroiditis has been reported in an 18-year-old woman 15 days after diagnosis of COVID-19, which improved after corticosteroids therapy (169).

## Discussion

### Limitations

Some limitations should be addressed. First, most of the clinical evidence was not systematic and based on case reports or case series without a long-term follow-up. Second, the sensitivity and specificity of different testing kits for SARS-CoV-2 infection were not well-validated, and the false positive or negative results could undermine our appraisal of the literature. Third, most of the reports were from patients with moderate to severe COVID-19 disease. It was impossible to determine the epidemiology of these manifestations in asymptomatic and mild COVID-19 cases. Fourth, overlapping COVID-19 features and concomitant medications could make it difficult to determine if these manifestations were immune-mediated. Fifth, it was difficult to assure whether direct cytopathic effect consequent to viral invasion or immune-mediated mechanisms were responsible for these manifestations. Lastly, the immunomodulators often used to treat these manifestations are also potential therapies for COVID-19. It was hard to know whether the immunomodulators exert their beneficial effect directly upon the immune mechanisms underlying these manifestations or indirectly through the alleviation of COVID-19 infection. However, there are no immunomodulating therapies that have consistently shown therapeutic efficacy toward SARS-CoV-2.

### Conclusions

**Figure 3:**
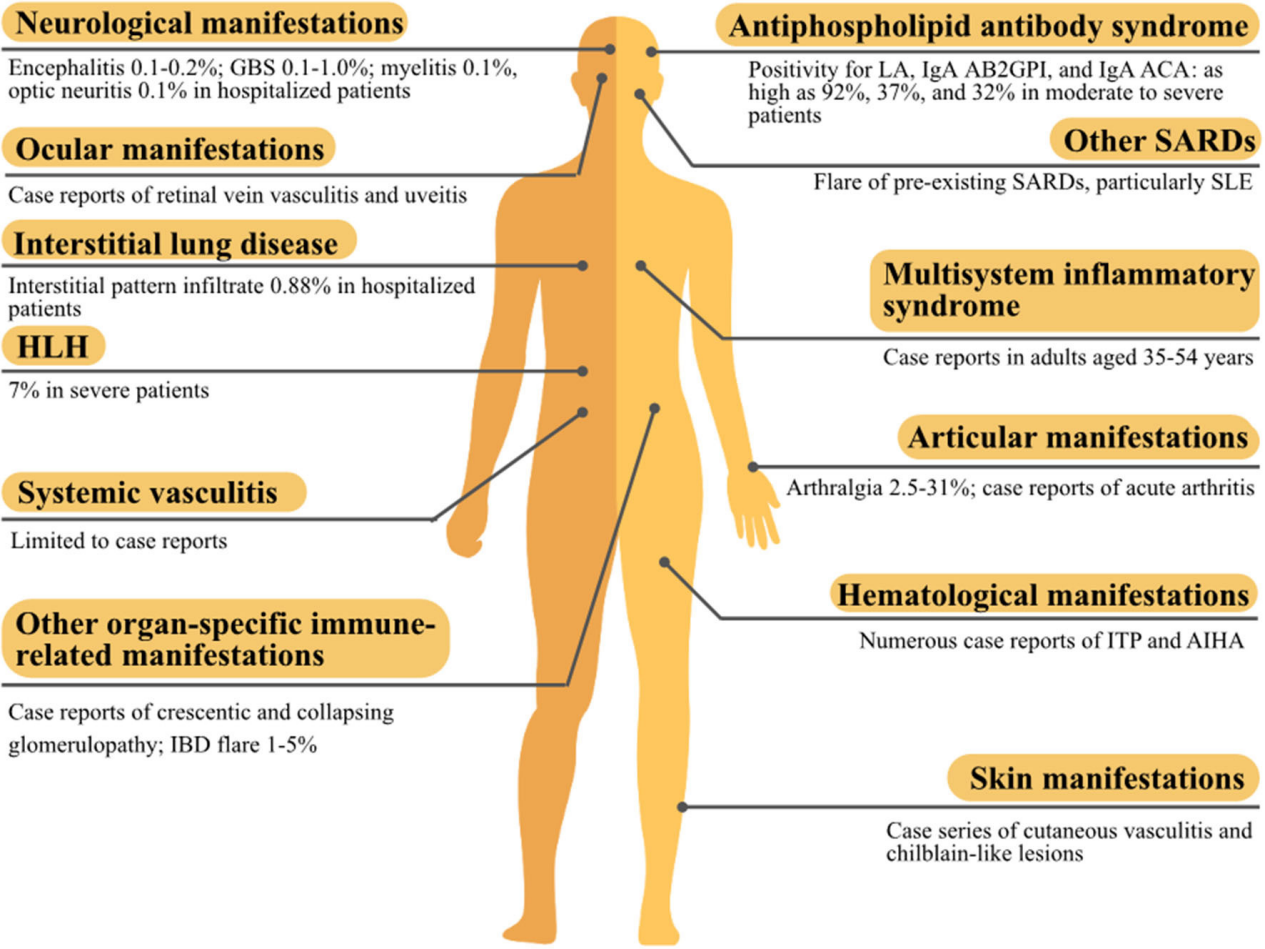
The summary of autoimmune and rheumatic manifestations associated with the coronavirus disease 2019 (COVID-19). AB2GPI, anti-β2glycoprotein I antibody; ACA, anticardiolipin antibody; AIHA, autoimmune hemolytic anemia; GBS, Guillain-Barré syndrome; HLH, hemophagocytic lymphohistiocytosis; IBD, inflammatory bowel disease; ITP, immune thrombocytopenic purpura; LA, lupus anticoagulant; SARD, systemic autoimmune rheumatic disease; SLE, systemic lupus erythematosus.

SARS-CoV-2 has a complex transcriptome and shares molecular similarities with human proteins, and its infection could generate various autoantibodies and cytokine storm, which form the basis for developing autoimmune and rheumatic manifestations. Accordingly, a variety of systemic or organ-specific manifestations have been reported to be associated with COVID-19 (summarized in Figure 3). Most of these manifestations have been reported in other microbial infections except for MIS-A. MIS-A shared some similarities with Kawasaki disease, but the distinct differences between the two entities made MIS-A more likely to be specific to SARS-CoV-2. In general, these manifestations were effectively treated in a strategy similarly used for patients without concomitant infection. Spontaneous recovery could happen but was uncommon, although expectant management was rarely undertaken in these patients. Based on the temporal relationship (sometimes delayed after COVID-19 infection resolves), well-known immune-mediated mechanisms, and treatment response to immunomodulators, these manifestations were probably consequences of the immune dysregulation caused by COVID-19 infection, particularly autoimmune cytopenia, cutaneous vasculitis, encephalitis, and GBS. But the evidence was still conflicting as regards to manifestations, such as APS, HLH, and MG. Herein, we provided a comprehensive overview of the evidence and literature concerning these rare but clinically significant manifestations; vaccine developers should take these findings into account in their vaccine design and post-marketing surveillance.

## Data Availability Statement

The raw data supporting the conclusions of this article will be made available by the authors, without undue reservation, upon request to the corresponding author.

## Author Contributions

K-TT, B-CH, and D-YC performed the literature search and retrieved relevant articles. K-TT and D-YC appraised the selected articles and drafted the manuscript. All authors made substantive intellectual contributions to the present study and approved the final manuscript.

## Conflict of Interest

The authors declare that the research was conducted in the absence of any commercial or financial relationships that could be construed as a potential conflict of interest.
