# Supplementary material for: Autoimmune and Rheumatic Manifestations Associated With COVID-19 in Adults: An Updated Systematic Review
Source: Front Immunol. 2021 Mar 12;12:645013. doi: 10.3389/fimmu.2021.645013 (PMC7994612; doi:10.3389/fimmu.2021.645013)
Supplement: Supplementary file 1 [file Data_Sheet_1.DOCX]

**Appendix 1.** Search strategies.

We searched PubMed and EMBASE on October 9, 2020. The search strategies were listed below.

**PubMed**

#1 COVID-19[Supplementary Concept] OR COVID*[tiab] OR 2019 novel coronavirus*[tiab] OR 2019-nCoV*[tiab] OR SARS-CoV-2*[tiab] OR coronavirus disease*[tiab] OR severe acute respiratory syndrome coronavirus 2 [Supplementary Concept] OR Wuhan coronavirus*[tiab]

#2 Autoimmune Diseases[MeSH] OR autoimmun* OR Autoimmunity[MeSH] OR Autoantibodies[MeSH] OR Autoimmune Diseases of the Nervous System[MeSH] OR Neurologic Autoimmun*[tiab] OR Nervous System Immune*[tiab]

# #3 autoinflammat*[tiab]

#4 Connective Tissue Diseases[MeSH] OR Connective Tissue*[tiab]

#5 Rheumatic Diseases[MeSH] OR Rheuma*[tiab]

#6 systemic lupus*[tiab] OR Libman Sacks*[tiab]

#7 Arthritis[MeSH] OR arthri*[tiab] OR Polyarthri*[tiab]

#8 Sjogren's Syndrome[MeSH] OR Sjogren*[tiab] OR Sicca*[tiab]

#9 Spondylarthropathies[MeSH] OR Spondyloarthro*[tiab] OR Spondylarthro*[tiab]

#10 Systemic Sclero*[tiab] OR Localized Sclero*[tiab] OR Morphea*[tiab] OR Linear Sclero*[tiab]

#11 Myositis[MeSH] OR Myosit*[tiab] OR Inflammatory Muscle*[tiab] OR Inflammatory Myopath*[tiab] OR Idiopathic Inflammatory Myo*[tiab]

#12 Hughes Syndrome*[tiab] OR Antiphospholipid*[tiab] OR Anti-Phospholipid*[tiab] OR Anti Phospholipid*[tiab]

#13 Vasculitis[MeSH] OR Vasculiti*[tiab] OR Angiiti*[tiab]

#14 Kawasaki*[tiab]

#15 Cryoglobulinemia[MeSH] OR Cryoglobulin*[tiab]

#16 Still's Disease, Adult-Onset[MeSH] OR Adult-Onset Still*[tiab] OR Adult Onset Still*[tiab]

#17 Lymphohistiocytosis, Hemophagocytic[MeSH] OR Hemophagocytic*[tiab] OR Reactive Hemophagocytic*[tiab] OR Infection-Associated Hemophagocytic*[tiab]

#18 Fibromy*[tiab] OR Fibrositi*[tiab] OR Secondary Fibromy*[tiab]

#19 Inflammatory Bowel Diseases[MeSH] OR Inflammatory Bowel*[tiab] OR Ulcerative Colitis[tiab] OR Crohn*[tiab]

#20 Psoriasis[MeSH] OR Psorias*[tiab]

#21 Uveitis[MeSH] OR Uveiti*[tiab]

#22 Lung Diseases, Interstitial[MeSH] OR Interstitial Lung*[tiab] OR Interstitial Pneumoni*[tiab]

#23 Glomerulonephritis[MeSH] OR Glomerulonephri*[tiab]

#24 Guillain*[tiab] OR Acute Inflammatory Demyelinating[tiab] OR Acute Inflammatory Polyneuropath*[tiab] OR Acute Inflammatory Polyradiculoneuropath*[tiab]

#25 Multiple Sclerosis*[tiab]

#26 Polyneuropathies[MeSH] OR Polyneuropath*[tiab] OR Acquired Polyneuropath*[tiab]

#27 Ocular Myasthenia*[tiab] OR Generalized Myasthenia*[tiab] OR Myasthenia*[tiab]

#28 Anemia, Hemolytic[MeSH] OR Hemolytic*[tiab] OR Acquired Hemolytic*[tiab]

#29 Thrombocytopenia[MeSH] OR Thrombocytopenia*[tiab] OR Thrombopenia*[tiab]

#30 #1 AND (#2 OR #3 OR #4 OR #5 OR #6 OR #7 OR #8 OR #9 OR #10 OR #11 OR #12 OR #13 OR #14 OR #15 OR #16 OR #17 OR #18 OR #19 OR #20 OR #21 OR #22 OR #23 OR #24 OR #25 OR #26 OR #27 OR #28 OR #29)

**EMBASE**

#1 ‘coronavirus disease 2019’/exp OR ‘2019-nCoV*’:ab,ti,kw OR ‘COVID*’:ab,ti,kw OR ‘nCoV 2019*’:ab,ti,kw OR ‘novel coronavirus*’:ab,ti,kw OR ‘Wuhan coronavirus*’:ab,ti,kw

#2 ‘autoimmune disease’/exp OR ‘auto-immun*’:ab,ti,kw OR ‘autoimmun*’:ab,ti,kw OR ‘autoantibody*’:ab,ti,kw

# #3 ‘autoinflammatory disease’/exp OR ‘autoinflammat*’:ab,ti,kw OR ‘auto-inflammat*’:ab,ti,kw

#4 ‘connective tissue disease’/exp OR ‘connective tissue*’:ab,ti,kw

#5 ‘rheumatic disease’/exp OR ‘rheuma*’:ab,ti,kw

#6 ‘systemic lupus*’:ab,ti,kw OR ‘lupus*’:ab,ti,kw

#7 ‘arthri’:ab,ti,kw OR ‘joint inflammat*’:ab,ti,kw

#8 ‘Sjogren*’:ab,ti,kw OR ‘sicca*’:ab,ti,kw

#9 ‘spondylarthropathy’/exp OR ‘spondyl*’:ab,ti,kw

#10 ‘scleroderm*’:ab,ti,kw OR ’systemic sclera*’:ab,ti,kw

#11 ‘myositis’/exp OR ‘idiopathic inflammatory myo*’:ab,ti,kw OR ‘inflammatory myo*’:ab,ti,kw OR ‘myositis*’:ab,ti,kw

#12 ‘Hughes syndrome*’:ab,ti,kw OR ‘antiphospholipid*’:ab,ti,kw OR ‘primary antiphospholipid*’:ab,ti,kw

#13 ‘vasculitis’/exp OR ‘vasculiti*’:ab,ti,kw OR ‘angiiti*’:ab,ti,kw

#14 ‘mucocutaneous lymph node syndrome’/exp OR ‘Kawasaki*’:ab,ti,kw OR ‘muco-cutaneous lymph node*’:ab,ti,kw OR ‘mucocutaneous lymph node*’:ab,ti,kw

#15 ‘cryoglobulinemia’/exp OR ‘cryoglobulin*’:ab,ti,kw

#16 ‘adult-Onset Still*’:ab,ti,kw

#17 ‘hemophagocytic syndrome’/exp OR ‘hemophagocytic*’:ab,ti,kw OR ‘haemophagocytic*’:ab,ti,kw

#18 ‘fibromyalgia’/exp OR ‘fibrositi*’:ab,ti,kw

#19 ‘inflammatory bowel disease’/exp OR ‘inflammatory bowel*’:ab,ti,kw

#20 ‘psoriasis’/exp OR ‘psorias*’:ab,ti,kw

#21 ‘uveitis’/exp OR ‘uveiti*’:ab,ti,kw OR ‘panuvei*’:ab,ti,kw OR ‘posterior uvei*’:ab,ti,kw

#22 ‘Interstitial lung disease’/exp OR ‘interstitial lung*’:ab,ti,kw

#23 ‘glomerulonephritis’/exp OR ‘glomerulonephri*’:ab,ti,kw

#24 ‘Guillain Barre syndrome’/exp OR ‘Guillain Barre*’:ab,ti,kw OR ‘Guillain-Barre*’:ab,ti,kw OR ‘Miller Fisher*’:ab,ti,kw OR ‘inflammatory acute polyradiculoneur*’:ab,ti,kw

#25 ‘multiple sclerosis’/exp OR ‘multiple sclerosis*’:ab,ti,kw

#26 ‘polyneuropathy’/exp OR ‘polyneuropath*’:ab,ti,kw

#27 ‘myasthenia gravis’/exp OR ‘myasthenia*’:ab,ti,kw

#28 ‘hemolytic anemia’/exp OR ‘hemolytic*’:ab,ti,kw OR ‘haemolytic*’:ab,ti,kw

#29 ‘thrombocytopenia’/exp OR ‘thrombocytop*’:ab,ti,kw

#30 #1 AND (#2 OR #3 OR #4 OR #5 OR #6 OR #7 OR #8 OR #9 OR #10 OR #11 OR #12 OR #13 OR #14 OR #15 OR #16 OR #17 OR #18 OR #19 OR #20 OR #21 OR #22 OR #23 OR #24 OR #25 OR #26 OR #27 OR #28 OR #29)
